# Evolutionary loss of peroxisomes – not limited to parasites

**DOI:** 10.1186/s13062-015-0101-6

**Published:** 2015-12-23

**Authors:** Vojtěch Žárský, Jan Tachezy

**Affiliations:** Department of Parasitology, Faculty of Science, Charles University in Prague, Viničná 7, 128 44 Prague, Czech Republic

**Keywords:** Peroxisome, Reduction, Parasitic helminths, *Oikopleura dioica*

## Abstract

**Background:**

Peroxisomes are ubiquitous eukaryotic organelles that compartmentalize a variety of metabolic pathways that are primarily related to the oxidative metabolism of lipids and the detoxification of reactive oxygen species. The importance of peroxisomes is underscored by serious human diseases, which are caused by disorders in peroxisomal functions. Some eukaryotic lineages, however, lost peroxisomes. These organisms are mainly anaerobic protists and some parasitic lineages including *Plasmodium* and parasitic platyhelminths. Here we performed a systematic *in-silico* analysis of peroxisomal markers among metazoans to assess presence of peroxisomes and peroxisomal enzymes.

**Results:**

Our analyses reveal an obvious loss of peroxisomes in all tested flukes, tapeworms, and parasitic roundworms of the order Trichocephalida. Intriguingly, peroxisomal markers are absent from the genome of the free-living tunicate *Oikopleura dioica*, which inhabits oxygen-containing niches of sea waters. We further map the presence and predicted subcellular localization of putative peroxisomal enzymes, showing that in organisms without the peroxisomal markers the set of these enzymes is highly reduced and none of them contains a predicted peroxisomal targeting signal.

**Conclusions:**

We have shown that several lineages of metazoans independently lost peroxisomes and that the loss of peroxisomes was not exclusively associated with adaptation to anaerobic habitats and a parasitic lifestyle. Although the reason for the loss of peroxisomes from *O. dioica* is unclear, organisms lacking peroxisomes, including the free-living *O. dioica*, share certain typical r-selected traits: high fecundity, limited ontogenesis and relatively low complexity of the gene content. We hypothesize that peroxisomes are generally the first compartment to be lost during evolutionary reductions of the eukaryotic cell.

**Reviewers:**

This article was reviewed by Michael Gray and Nick Lane.

**Electronic supplementary material:**

The online version of this article (doi:10.1186/s13062-015-0101-6) contains supplementary material, which is available to authorized users.

## Open Peer Review

Reviewed by Michael Gray and Nick Lane. For the full reviews, please go to the Reviewers' comments section.

## Background

Peroxisomes are single membrane-bound organelles that proliferate by fission, although it has been shown that peroxisomes can emerge *de novo* from the endoplasmic reticulum [1]. Peroxisomes participate in a variety of metabolic functions, such as the reactive oxygen species detoxification, long-chain fatty acid beta-oxidation, plasmalogen synthesis, amino acid degradation, and purine metabolism [2]. The diversity of peroxisomal functions is well exemplified by atypical peroxisomes, termed glycosomes, which compartmentalize the first seven enzymes of glycolysis and which are indispensable for the survival of *Trypanosoma brucei,* the causative agent of sleeping sickness [3]. Other types of peroxisomes with highly specialized roles have been described, such as glyoxysomes in plants and Woronin bodies in filamentous ascomycetes [4].

A unique group of proteins referred to as peroxins (Pexs) is required for peroxisome biogenesis and protein import. Peroxins mediate the post-translational import of folded proteins bound to cofactors or even of protein complexes [5]. Enzymes destined for the peroxisomal matrix are recognized by the specific cytosolic receptors Pex5 and Pex7. Pex5 recognizes the peroxisomal targeting signal 1 (PTS1), which is composed of a canonical Ser-Lys-Leu tripeptide at the extreme C-terminus with common deviations of the canonical sequence [6]. Some other proteins carry a nonapeptide motif near the N-terminus termed PTS2, which is recognized by Pex7 [7].

A protein (cargo) carrying the PTS1 sequence is first recognized by soluble Pex5, which then interacts with the peroxisomal membrane proteins Pex14 and Pex13, which leads to a translocation into the peroxisomal lumen [8–10]. Pex5 is then either monoubiquitinated by Pex10 and Pex12 E3 ubiquitin ligases or polyubiquitinated by the Pex2 E3 ubiquitin ligase [11, 12]. The monoubiquitinated Pex5 is recycled to the cytoplasm by Pex1 and Pex6, both of which carry two ATPase associated with diverse cellular activities (AAA) [13] and which in mammals are recruited to the membrane by the Pex26 protein [14]. In *Saccharomyces cerevisiae*, the function of Pex26 is carried out by an unrelated protein Pex15, which is specific to yeast [15]. Alternatively, polyubiquitinated Pex5 is degraded by the proteasome.

Hydrophobic proteins targeted to the peroxisomal membrane are typically recognized by the cytosolic receptor Pex19, which binds to the peroxisomal membrane proteins Pex3 and Pex16 [16, 17]. Subsequently, the cargo protein is inserted into the peroxisomal membrane. Alternatively, some peroxisomal membrane proteins are first inserted into the ER and are then transported to the peroxisomal membrane via a process that depends on Pex19 and Pex3 [18]. In some organisms (e.g. in *Saccharomyces cerevisiae*) Pex16 is absent [19].

**Fig. 1:**
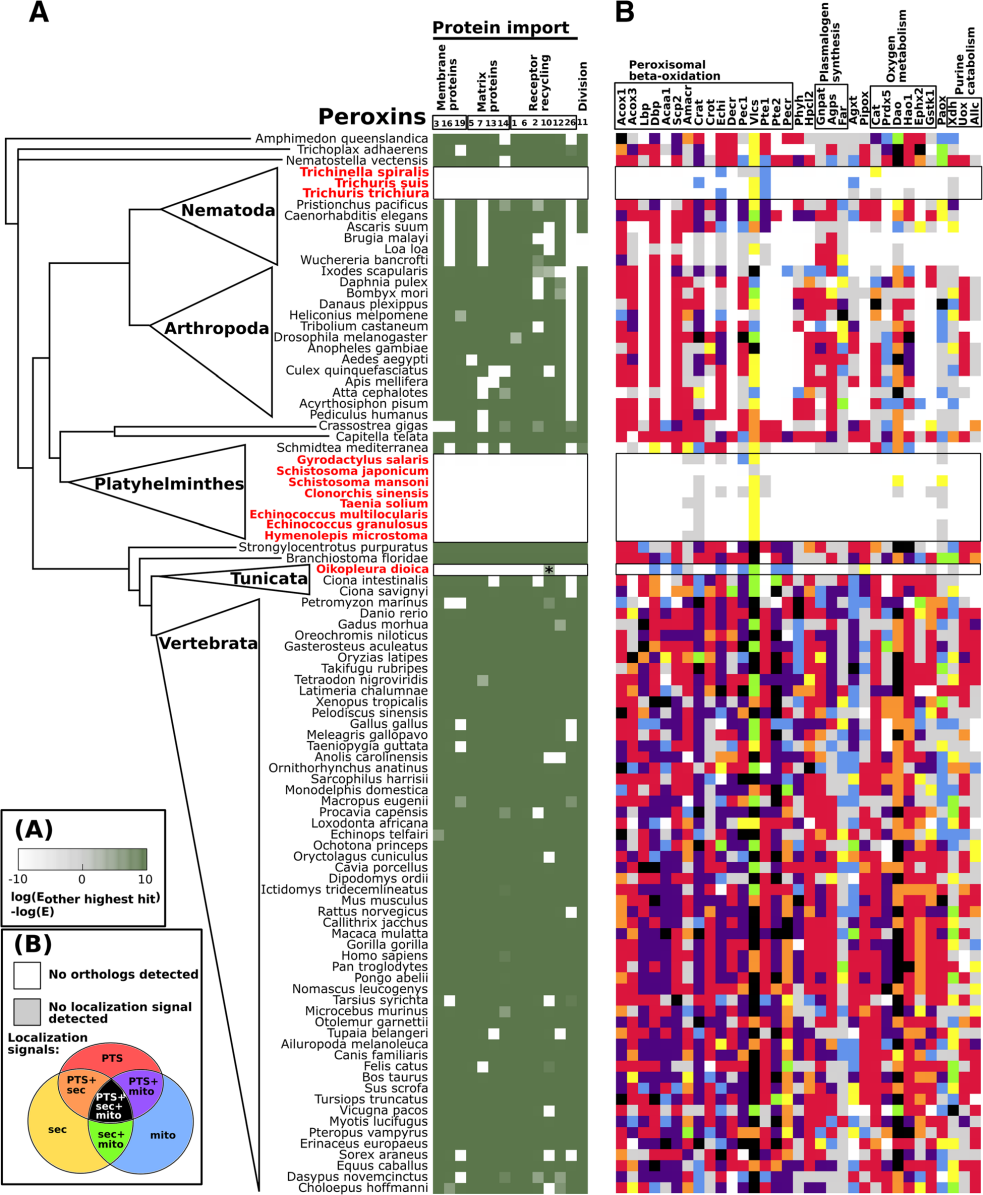
Distribution of peroxins (A), and possible peroxisomal enzymes (B) in metazoan genomes. Organisms lacking peroxins are highlighted in red. **a** Identification of peroxins. The green squares indicate identification with high confidence. The identification of Pex10 in the *Oikopleura dioica* genome with lower confidence (marked by an asterisk) was rejected as a false-positive based on phylogenetic analysis (Figure S1). **b** Identification of putative peroxisomal enzymes and enyzmes of mitochondrial beta-oxidation. Predicted subcellular localization is indicated in the following colors: grey, no targeting signal detected; red, peroxisomal targeting sequence type 1 or 2 (pts); blue, mitochondrial localization signal (mito); and yellow, secretory pathway signal peptide (sec). Combinations of several different localization signals are shown as follows: violet, pts and mito; orange, pts and sec; green, sec and mito; and black, pts, mito and sec. (Acox1 - Acyl-CoA oxidase1, Acox3 - Acyl-CoA oxidase 3, Lbp - L-bifunctional protein, Dbp - D-bifunctional protein, Acaa1 - Peroxisomal beta-ketothiolase 1, Scp2 - Peroxisomal beta-ketothiolase 2, Amacr - Alpha-methylacyl-CoA racemase, Crat - Carnitine acetyltransferase, Crot - Carnitine octanoyltransferase, Ech1 - Enoyl Coenzyme A hydratase 1, Decr - Peroxisomal 2,4-dienoyl-CoA reductase 2, Pec1 - Peroxisomal 3,2-trans-enoyl-CoA isomerase, Vlcs - Very-long-chain acyl-CoA synthetase, Pte1 - Acyl-CoA thioesterase 2, Pte2 - Acyl-CoA thioesterase 1B, Phyh - Phytanoyl-CoA 2-hydroxylase, Hpcl2 - 2-Hydroxyphytanoyl-CoA lyase, Gnpat - Dihydroxyacetone phosphate acyltransferase, Agps - Alkyldihydroxyacetone phosphate synthase, Far - Fatty acyl-CoA reductase 2, Agxt - Alanine:glyoxylate aminotransferase, Pipox - Peroxisomal sarcosine oxidase/L-pipecolate oxidase, Cat - Catalase, Prdx5 - Peroxiredoxin V, Dao - d-amino acid oxidase, Hao1 - Hydroxyacid oxidase 1, Ephx2 - Epoxide hydrolase, Gstk1 - Glutathione S-transferase class Kappa, Paox - N1-acetylspermine/spermidine oxidase, Xdh - Xanthine dehydrogenase, Uox - Uricase, Allc – Allantoicase)

As the enzymatic content of peroxisomes is known to vary considerably among species or even different tissues, the components of the peroxisomal protein import system are the most reliable peroxisomal markers. A core set of at least 13 peroxins is common to the main eukaryotic lineages (Pex1,2,3,5,6,7,10,11,12,13,14,16,19, Fig. 1a); thus, these proteins were probably present in the last common ancestor of eukaryotes [20, 21].

A novel trafficking route between mitochondria and peroxisomes, which is mediated by the mitochondria-derived vesicles (MDV) was recently described [22]. Specific subset of MDVs that contain mitochondrial-anchored protein ligase (MAPL) was shown to fuse with a subpopulation of peroxisomes. However the function of MDVs in regard to peroxisomes is unknown and none of the discovered components of MDVs can be considered to be a specific peroxisomal marker. Thus, we didn't include these components to our dataset.

The essential role of peroxisomes is underlined by the increasing list of diseases that are associated with disorders of peroxisome biogenesis or even the dysfunction of a single peroxisomal enzyme [23]. However, some unicellular eukaryotes are able to live without peroxisomes. These eukaryotes includes parasitic protists that live in oxygen-poor environments, such as *Giardia intestinalis*, *Entamoeba histolytica* and *Trichomonas vaginalis*, and the loss of peroxisomes has also been described in intracellular parasites of the Apicomplexa (e.g., *Plasmodium falciparum*, *Cryptosporidium parvum*) and Microsporidia (e.g., *Encephalitozoon cuniculi*) [4, 21]. However peroxisomes were identified in an apicomplexan *Toxoplasma gondii* [24]. More recently, the loss of many genes associated with peroxisomes was observed in the genomes of certain parasitic helminths, including tapeworms and flukes and a loss of peroxisomes in those lineages was suggested [25, 26]. Here, we executed a large-scale analysis of metazoan genomes to assess the presence of the systems required for the biogenesis and metabolic function of peroxisomes. Our results revealed the unexpected loss of peroxisomal functions and peroxisomes in metazoans including not only parasitic species but also, surprisingly, an aerobic free-living organism.

## Results and discussion

### Loss of peroxins and peroxisomes

To assess the presence of peroxisomal components, we collected an extensive dataset of the predicted proteomes of 111 metazoans based on completed or advanced genome sequencing projects (see Additional file 1: Table S1). We then assessed the presence or absence of 14 peroxins conserved in metazoans [21] (see Additional file 2: Table S2) by assigning the protein sequences to the evolutionary genealogy of genes: Non-supervised Orthologous Groups (eggNOG) database of orthologous groups, which was constructed from representative metazoan sequences [27]. For homology analysis, we used a highly sensitive profile-hidden Markov model (HMMER) search algorithm [28].

We found a broad set of peroxins in most metazoans (Fig. 1a). However, not all of these metazoans contained a complete set of peroxins. Peroxins such as Pex26 appeared to be repeatedly lost during evolution, as we observed the lack of this protein in nematodes and other invertebrates. We also observed the loss of the PTS2-binding protein Pex7 in all nematodes which is in agreement with lack of the PTS2 pathway in *Caenorhabditis elegas* and other lineages [29–31]. Some other peroxins might have diverged beyond recognition by sequence analysis, as was proposed for a hypothetical *Trypanosoma brucei* Pex3 [32].

The extreme case of the reduction of the peroxisomal components is the complete loss of peroxisomes. It has been established experimentally that the loss of certain peroxins that are essential for the peroxisome biogenesis, such as Pex3 and Pex19, leads to the complete loss of peroxisomes [1]. Thus, we interpreted absence of these peroxins as evidence for the loss of peroxisomes per se in the examined organisms. We did not identify any peroxins in the genome of several lineages of parasitic helminths: flukes (*Schistosoma japonicum*, *S. mansoni*, and *Clonorchis sinensis*), tapeworms (*Taenia solium*, *Echinococcus multilocularis*, *E. granulosus*, and *Hymenolepis microstoma*) and a monogenean (*Gyrodactylus salaris*) as was described previously [25, 26]; all of these species belong to the Neodermata group of parasitic flatworms. Furthermore, we observed the independent loss of peroxins in the parasitic roundworms of the order *Trichocephalidae*, including *Trichinella spiralis* and the whipworms *Trichuris trichiura* and *Trichuris suis*. Most surprisingly, in addition to parasites, we did not identify any convincing orthologs of peroxins in the free living tunicate *Oikopleura dioica*. This organism belongs to the class *Appendicularia*, occupies the pelagic zone of the world's oceans, and exhibits a high rate of oxygen consumption [33] as well as several peculiar features: (i) its genome is extremely small (approximately 75 Mb), (ii) it has a short generation time (as short as 24 hours [34]), and (iii) adults of this species have a simplified body structure that resembles the tadpole-like larvae of most tunicates [35, 36]. Interestingly, the related marine tunicates *Ciona intestinalis* and *Ciona savignyi* of class *Ascidiacea* harbor a regular set of peroxins. Our analysis revealed only one possible hit of Pex10, based on its zinc-finger domain, in *O. dioica*. However, the predicted protein lacks a typical N-terminal hydrophobic domain, and phylogenetic analysis of the zinc finger domain revealed that it is likely a false-positive hit as it forms a monophyletic clade with mammalian NHLRC1 proteins, while the Pex10 sequences form a distinct clade (Additional file 3: Figure S1).

### Peroxisomal pathways

To further corroborate our findings, we predicted the presence of peroxisomal matrix-resident enzymes based on predicted PTS1 and PTS2 motifs [7, 37]. For the detection of the signals we used PTS1/2 amino acid motifs according to the PSORT II software [38] and we also included sequences of mammalian PTS1, which do not comply with these motifs. We focused on the components of main peroxisomal functional pathways, including oxygen metabolism, beta-oxidation, plasmalogen biosynthesis, amino acid metabolism, and purine catabolism [2] (Fig. 1b, Additional file 4: Table S3). We further predicted mitochondrial and secretory signal peptides using TargetP software [39].

In the metazoans lacking Pexs identified above, most of the conserved peroxisomal matrix enzymes have been lost, and the few enzymes that are retained contained no apparent peroxisomal localization signals (Fig. 1b). Pathways that are exclusively found in organisms harboring peroxisomes include peroxisomal beta-oxidation (acyl-CoA oxidase1/Acox1, L-bifunctional protein/Lbp, peroxisomal beta-ketothiolase 1/Acaa1, and peroxisomal 2,4-dienoyl-CoA reductase 2/Decr) and plasmalogen synthesis (fatty acyl-CoA reductase 1/Far1, dihydroxyacetone phosphate acyltransferase/Gnpat and alkyldihydroxyacetone phosphate synthase/Agps). Interestingly, a gene coding for the typical peroxisomal enzyme catalase was detected in the genome of *Trichinella spiralis*; the predicted protein, however, lacks a PTS signal but has an N-terminal signal peptide instead.

The prediction of targeting signals in the putative peroxisomal proteins revealed that enzymes typically possess either peroxisomal or mitochondrial localization signals or both, indicating a metabolic interdependence between peroxisomes and mitochondria and, possibly, the dual targeting of some enzymes (Fig. 1b). This is a well known phenomenon exemplified by e.g. the human isoform of carnitine acetyltransferase (Crat), which is important for both the mitochondrial and peroxisomal beta-oxidation in mammalian cells, and contains a mitochondrial localization signal at the N-terminus and a typical PTS1 sequence at the C-terminus [40].

### Loss of peroxisomes and the genome complexity

**Fig. 2:**
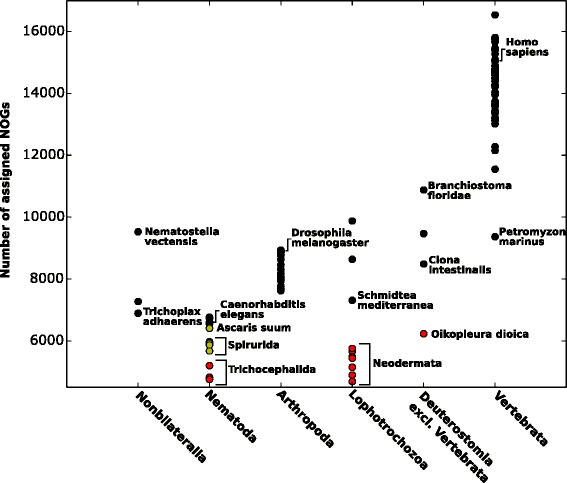
Number of unique orthologous groups in the metazoan eggNOG database that were assigned to genomes. Organisms were sorted into crude taxonomic groups. For a complete list, see Table S1. Organisms lacking peroxins are shown in red. Parasitic nematodes harboring peroxisomes are represented by yellow circles.

The identified metazoan lineages lacking peroxisomes have very different phylogenetic positions and lifestyles: *Trichinella* and *Trichuris* develop in vertebrate cells and/or host tissues; schistosomes and tapeworms first develop in intermediate hosts and then reside in the bloodstream and the intestine of their definitive hosts, respectively; *Gyrodactylus salaris* is an ectoparasite of fish; and *Oikopleura* is a marine filter feeder. However, in ecological terms, all of these species share certain typical r-selected traits: high fecundity, early maturation, and simplified ontogenesis. To assess the relative complexity of their genomes and to evaluate the loss of peroxisomes in the genomic context, we quantified unique orthologous groups of proteins that were detected in the predicted proteomes (Fig. 2; Additional file 1: Table S1). It is apparent that lineages lacking peroxisomes contain a markedly reduced repertoire of conserved metazoan orthologs compared to their relatives harboring peroxisomes. Therefore, the loss of peroxisomes clearly accompanied the reductive evolution of the respective metazoan lineages, as reflected by genome shrinkage and gene loss.

## Conclusions

In addition to the known loss of peroxisomes in parasitic flatworms (Neodermata) we observed the loss of peroxisomes in parasitic roundworms of the order Trichocephalida and most intriguingly in a free-living tunicate *Oikopleura dioica*. The loss of peroxisomes from Neodermata and Trichocephalida likely reflects their adaptation to an obligate parasitic lifestyle, which is specifically associated with the adaptation to anaerobiosis of some of the life stages and/or the reduced need for lipid synthesis and turnover. However, not all parasitic worms have lost peroxisomes, as we detected the presence of peroxisomal markers in the genomes of some helminths, such as *Ascaris suum*. The presence of peroxisomes in these organisms can be explained by aerobic metabolism during L1 and L2 larval stages of *Ascaris*, which live in an external environment in eggs, and in L3 larvae that migrate from the intestines to the lungs via the liver after egg ingestion. Only L4 larvae and adults live in the oxygen-poor environment of intestines and utilize anaerobic metabolism. In this context, the absence of peroxisomal markers in *Oikopleura dioica*, a free-living tunicate that inhabits oxygen-containing niches of sea waters and that exhibits a high rate of oxygen consumption [33], is highly unexpected. Similar to other aerobes, *O. dioica* possesses classical mitochondria that utilize oxygen and oxidize fatty acids for ATP synthesis. Why might this organism have lost peroxisomes? We hypothesize that the loss of peroxisomes is associated with the reduction of genomic content and generally with a shift towards r-selected traits, which is typical for parasitic organisms. However, there might be other advantages of peroxisomal loss, e.g., rendering the organisms resistant to xenobiotics that become activated in the peroxisomal lumen in response to frequent redox reactions [41].

Comparative genomics shows that most of the typical cell compartments were present in the common eukaryotic ancestor [42]. However there are known cases where these compartments were radically functionally and/or structurally reduced, as the divergent anaerobic mitochondria of *Giardia intestinalis* called mitosomes [43], fragmented ER of *Entamoeba histolytica* [44], or absence of structured Golgi in several protist lineages [45, 46]. Still in all these cases the organelle and the apparatus for its biogenesis are to various extents conserved and retained. On the contrary it has been shown that peroxisomes have been lost on several occasions, not only in parasitic and anaerobic lineages. We thus further hypothesize that peroxisomes are generally the first conserved eukaryotic compartment to be lost during reductive evolution of the cell.

As the loss of peroxisomes was repeatedly observed in various metazoan lineages, it is likely that new lineages lacking peroxisomes will emerge and that new genomic and functional data will provide additional information to reveal the circumstances in which selection pressure resulted in the loss of peroxins and, consequently, peroxisomes.

## Methods

### Orthologous group assignment

Orthologous groups of metazoan proteins were extracted from the eggNOG database, ver. 3 [27]. Predicted metazoan protein sequences were searched against the HMM profiles created from the alignments of eggNOG groups using HMMER software [28]. Hits displaying an e-value greater than 1e-3 were regarded as false positives. The hits were scored based on their e-value (Ehit) and the next highest hit e-value (Enext) as follows: Score = log(Enext)-log(Ehit). Thus, only the best hits displayed a positive score.

### Subcellular localization prediction

N-terminal targeting sequences were predicted using TargetP software with default settings [39]. The peroxisomal targeting signals were predicted using the following regular expressions: PTS1: '[SAC][KRHN][LIM]' or 'ASL', 'THL', 'LKL', 'SKV', 'KKL', 'SQL', or 'PRL' at the C-terminus; PTS2: '[RK][LVIQ]..[LVIHQ][LSGAK]..[HQ][LAF]' within the first 100 amino acids [2, 38].

### Phylogenetic analysis

Homologs of *O. dioica* GSOIDT00013970001 were retrieved from the SwissProt database using BLAST [47]. Sequences were aligned using MAFFT [48], trimmed and the phylogeny was constructed with Phyml [49] using the WAG substitution matrix.

## Reviewers' comments

### Reviewer's report 1 (Michael Gray, Dalhousie University, Canada)

**Summary:** This manuscript describes the results of a comprehensive survey to detect peroxisome gene markers (encoding peroxins and other characteristic peroxisomal proteins) in complete or substantially complete animal genome sequences. The results extend previous results that suggested the apparent absence of peroxisomes in certain parasitic (anaerobic) helminths. Unexpectedly, the authors also report the absence of peroxisomal markers from the genome of a free-living (and oxygen-consuming) tunicate. This study is straightforward in its execution and in the results obtained. The authors employed rigorous methods of gene identification, including HMM profile searches, to ensure that peroxisomal markers being sought were not missed. The results are clear-cut: Fig. 1 is especially effective in summarizing the main message. The authors point out that peroxisomal loss in animals correlates with a gene content of relatively low complexity (Fig. 2), from which they argue that peroxisomes may be the first compartment to be lost during evolutionary down-sizing of the eukaryotic cell.

**Author's response:** We thank the reviewer for his appreciation of our work.

**Recommendations:** I have no substantive criticisms of the work, which overall is novel and will be of interest to a wide readership. The results emphasize the importance of taking a comprehensive and rigorous approach to questions of presence/absence of genes/organelles before drawing firm evolutionary conclusions. Extension of this work to other groups of eukaryotes, as sufficient genome data become available, should certainly be done: fungi are an obvious first choice, but eukaryotic microbes (protists) should also be examined by the same approach.

**Author's response:** We agree with the reviewer that our analysis of peroxisomal markers should be extended to a wider range of eukaryotes in the future. In fact, we performed the preliminary analysis of peroxisomal markers across major eukaryotic lineages, however, we didn't find any novel losses of peroxisomes except these in anaerobic protists, some apicomplexans and microsporidians that are mentioned in the Background section. Nevertheless, we can expect more variation in individual peroxins (losses and gains) as well as more functional diversity in various lineages of unicellular eukaryotes than in metazoans. This has been shown for example in the case of mitochondria that displayed considerably higher level of diversity in unicellular eukaryotes in comparison to metazoans. From this point of view, described losses of peroxisomes within metazoans including *Oikopleura dioica* are really unexpected and thus we would prefer the paper to be focused on the analysis of metazoans to keep the publication more straightforward.

**Minor issues:** While reading through the manuscript, I did encounter a few grammatical issues that I flagged in the attached PDF. In particular, the term ‘r-selected traits’ should be defined for the benefit of readers who will be unfamiliar with the concept.

**Author's response:** We edited the manuscript according to the reviewer's recommendations. We also explained the concept of 'r-selected traits'.

### Reviewer's report 2 (Nick Lane, UCL, United Kingdom)

**Summary:** This is an interesting and short phylogenetic paper, which shows that peroxisomes have almost certainly been lost from various lines of metazoans over evolutionary time, not only in parasitic flukes, tapeworms and roundworms, but also in some free-living tunicates that inhabit oxygenated waters. The findings extend and strengthen earlier observations of loss of peroxisomes. These results are of interest in themselves, but could probably benefit from a little more discussion about their evolutionary significance.

**Author's response:** We thank the reviewer for his appreciation of our work.

**Recommendations:** The paper is clear and crisp, with little extraneous discussion. I would be happy to see it published more or less as it is. In my view, the paper would be stronger if it had included an ultrastructural study - I appreciate that it is hard to show the absence of structures by EM, but while the genetic dataset is fairly convincing evidence (and even if not, certainly worth publishing) the paper would be stronger still if it had included immunogold-labeling in the groups with or without peroxisomes, ER and so on. As Shakespeare said, give me the ocular proof. But I appreciate that this is not easily done, and I reiterate that the paper could be published as it is.

**Author's response:** While preparing the paper we faced an obvious principal problem as proving a nonexistence of anything is usually difficult and to some extend dubious. We were considering the electron microscopy to confirm the absence of peroxisomes, which are traditionally visualized by detecting the catalase activity using 3,3′ diaminobenzidine tetrahydrochloride (DAB). However e.g. in *Plasmodium falciparum* peroxidase active organelles were discovered although there is a well supported consensus that there are no peroxisomes present and interpretation of such results is difficult [50]. Thus, we decided to rely on the genomic data which shows a comprehensive set of coding genes. In organisms with predicted loss of peroxisomes we carefully checked for possible peroxin homologs in the predicted protein sequences, genome sequences translated in all 6 frames and in the transcriptomic data.

**Recommendations:** I would have appreciated a little more discussion of the evolutionary significance of the findings. For example, de Duve long argued that peroxisomes had an endosymbiotic origin, albeit with declining evidence over the last decade. I wondered if the authors have anything to say about the ease of loss of peroxisomes in relation to hydrogenosomes or mitosomes as organelles. How do they disappear? Is their loss linked with changes in the ER? These are all questions not directly addressed in the paper, and so perhaps not relevant, but some short discussion of these issues would not go amiss. I find it interesting that it seems to be so easy to lose peroxisomes but far less easy to lose extra membranes from around secondary or tertiary chloroplasts, or to lose mitochondria completely, or indeed ER. Why the peroxisomes and maybe Golgi? Does it relate to nuclear membrane dissolution during mitosis or meiosis?

**Author's response:** The origin of peroxisomes is a matter of discussion since their discovery by Christian de Duve. He proposed an endosymbiotic origin of the organelle [51, 52] that is supported mainly by a post-translational import of proteins to the peroxisomes, and the biogenesis of new peroxisomes by fission of pre-existing peroxisomes, the features known for endosymbiotic organelles such as mitochondria. Later it was, however, discovered that peroxisomes can arise de novo from the endoplasmic reticulum by knock-in of essential peroxin gene into peroxisome-lacking mammalian cells and yeast mutants and that peroxisomes are formed de novo also in normal cells [1, 53]. Some of the components of the peroxisomal protein import machinery are also homologous to the ERAD (Endoplasmic-reticulum-associated protein degradation) pathway of the endoplasmic reticulum [20]. These observations support an alternative hypothesis of endogenous origin of peroxisomes.

Our finding of relatively common evolutionary loss of peroxisomes is in a contrast with primary endosymbiotic organelles (mitochondria, chloroplasts) which are known to undergo reductions but to our knowledge there are no known cases of their complete loss. What is a reason for such a difference? Based on Blobel's idea of membranes heredity [54], we can speculate that ER represent a source of membranes for peroxisome formation, which allows high dynamics of peroxisomes in respect of their size and number, *de novo* formation and eventually loss upon environmental conditions. However, mitochondrial membranes cannot arise de novo and cells may have mechanisms to prevent such loses. The other reason might be that none of peroxisomal functions is really essential, whereas mitochondria possess iron-sulfur cluster assembly (ISC) machinery that seems to be indispensable for all viable cells (of course we can discuss exceptions when ISC machinery is replaced by other systems). We do not think that easier loss of peroxisomes is related to nuclear membrane dissolution during mitosis or meiosis because e.g. Trichomonad mitosis is the closed mitosis without dissolution of the nuclear membrane, but they do not have peroxisomes.

**Recommendations:** Fig. 2 is interesting but seems a bit minimal in terms of identifying specific groups. For example, where is *C. intestinalis* and other groups mentioned in the text? I appreciate that this information is available in Table S1, but that is a frustrating format to compare information. A few more labels on Fig. 2 showing key comparators mentioned in the text would be valuable for those who are interested but don't want to spend a long time wading through the SI.

**Author's response:** We added labels in Fig. 2 for selected model species and organisms related to the proposed lineages missing peroxisomes.

**Minor issues:** I was uncertain about whether the presence/absence data for peroxins etc. are based on proteomes (as stated on page 5) or whether some data are based on full genome sequences.

**Author's response:** Our analysis was based exclusively on the *in silico* predicted proteomes from genomic data. When the loss of peroxisomes was suspected we further checked the available genomic and transcriptomic data. We clarified this issue at pages 5 and 7 in the text.

**Minor issues:** Many parasites have different phases of their life cycle, and I imagine (though don't know much about it) that their proteomes could differ significantly at different stages. It wouldn't be surprising to find that some parasites have peroxisomes at some stages of their life cycle but not others. This was not discussed at all, or at least I didn't notice it; and is not clear to me from the data.

**Author's response:** It is likely that in the parasitic helminths with peroxisomes the significance of peroxisomal functions, and the size and quantity of peroxisomes will be altered during the life cycle as this is well described in the case glycosomes of trypanosomatids [55]. However we are not aware of any such data in the case of analyzed parasitic helminths. Moreover, our data are based on *in silico* predictions of proteins, not on proteomic studies that could be affected by variations in proteomes.

**Minor issues:** I wondered if there are any known non-canonical pathways of peroxisome targeting that do not involve peroxins. Plainly these are missing, and I accept the conclusion that the peroxisomes have most likely been lost, given that other aspects of peroxisome metabolism are also missing. Even so, these details could at least be mentioned in the discussion.

**Author's response:** Indeed a novel trafficking route between mitochondria and peroxisomes has been described. We mention this pathway in the Background section.

**Minor issues:** How does *Oikopleura dioica* survive in oxygenated waters without peroxisomes? This is genuinely an interesting finding, and it might be that r-selection is indeed sufficient to explain the loss. It would be good to know how large population sizes (Ne) tend to be, and developmental time compared with related tunicates such as *C. intestinalis*, which do have peroxisomes. To a degree this probably correlates with NOG data in Fig. 2, but not entirely. I also wondered whether *O. dioica* had other mechanisms of oxygen detoxification, such as an alternative oxidase or uncoupling proteins. This is probably reasonably easy to check in their proteomic data and would be worth commenting on.

**Author's response:** The differences between *Ciona intestinalis* (*Ascidiacea*) and *Oikopleura dioica* (*Appendicularia*) are truly striking. The adults of *C. intestinalis* are up to 15 cm long sessile sea squirts and their life cycle takes about 2 months. On the other hand the adults of *O. dioica* are pelagic tunicates of size between 0.5 and 1mm that resemble the tadpole-like larva of ascidiaceans with a generation time as short as 24 hours [34]. Comparison of two distinct haplotypes of *O. dioica* revealed a high estimate of population mutation rate, which is consistent with large effective population size and/or high mutation rate per generation [56].

Interestingly, our searches for alternative oxidases (AOX) revealed homologs of AOX in the genomes of *C. intestinalis* and *C. savignyi*. Counterintuitively we did not identify AOX in the genome of *O. dioica*. Furthermore, unlike most metazoans, *O. dioica* possesses only a single uncoupling protein (UCP4).

## Additional files

Additional file 1: Table S1. Table of genomes used in the analysis with the count of assigned orthologous groups (NOGs). (XLS 59 kb) Additional file 2: Table S2. Table of peroxins identified. (XLS 677 kb) Additional file 3: Figure S1. Phylogeny of Pex10 and other zinc-finger domain containing proteins showing, that *O. dioica* GSOIDT00013970001 sequence isn't monophyletic with eukaryotic Pex10 sequences. Bootsrap supports are shown. (PDF 351 kb) Additional file 4: Table S3. Table of putative peroxisomal enzymes and the prediction of their localization. (XLS 7288 kb)

## Abbreviations

PTS: Peroxisomal targeting signa
Pex: Peroxin
ER: Endoplasmic reticulum
L1-4: Larval stages
ATP: Adenosine triphosphate
Acox1: Acyl-CoA oxidase 1
Acox3: Acyl-CoA oxidase 3
Lbp: L-bifunctional protein
Dbp: D-bifunctional protein
Acaa1: Peroxisomal beta-ketothiolase 1
Scp2: Peroxisomal beta-ketothiolase 2
Amacr: Alpha-methylacyl-CoA racemase
Crat: Carnitine acetyltransferase
Crot: Carnitine octanoyltransferase
Ech1: Enoyl Coenzyme A hydratase 1
Decr: Peroxisomal 2,4-dienoyl-CoA reductase 2
Pec1: Peroxisomal 3,2-trans-enoyl-CoA isomerase
Vlcs: Very-long-chain acyl-CoA synthetase
Pte1: Acyl-CoA thioesterase 2
Pte2: Acyl-CoA thioesterase 1B
Phyh: Phytanoyl-CoA 2-hydroxylase
Hpcl2: 2-Hydroxyphytanoyl-CoA lyase
Gnpat: Dihydroxyacetone phosphate acyltransferase
Agps: Alkyldihydroxyacetone phosphate synthase
Far: Fatty acyl-CoA reductase 2
Agxt: Alanine:glyoxylate aminotransferase
Pipox: Peroxisomal sarcosine oxidase/L-pipecolate oxidase
Cat: Catalase
Prdx5: Peroxiredoxin V
Dao: d-amino acid oxidase
Hao1: Hydroxyacid oxidase 1
Ephx2: Epoxide hydrolase
Gstk1: Glutathione S-transferase class Kappa
Paox: N1-acetylspermine/spermidine oxidase
Xdh: Xanthine dehydrogenase
Uox: Uricase
Allc: Allantoicase
