# Supplementary figures and images for: Evolutionary loss of peroxisomes – not limited to parasites
Source: Biol Direct. 2015 Dec 23;10:74. doi: 10.1186/s13062-015-0101-6 (PMC4690255; doi:10.1186/s13062-015-0101-6)

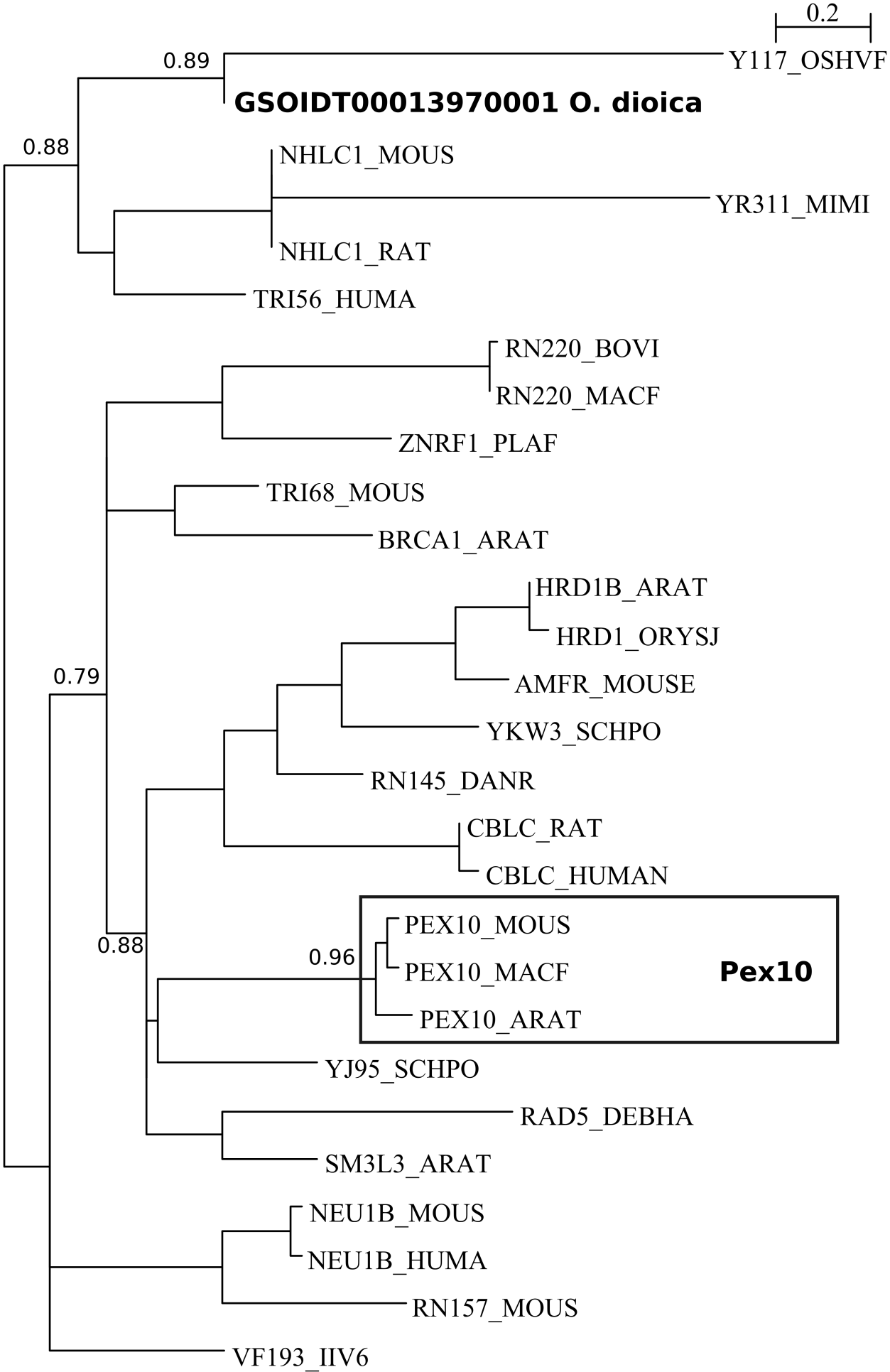

Supplement: Additional file 3: Figure S1. — Phylogeny of Pex10 and other zinc-finger domain containing proteins showing, that O. dioica GSOIDT00013970001 sequence isn't monophyletic with eukaryotic Pex10 sequences. Bootsrap supports are shown. (PDF 351 kb) [file 13062_2015_101_MOESM3_ESM.pdf]
